# The Secretome of iPSC-Derived Mesenchymal Stem Cells Alters Glial and Vascular Responses in SCI-Relevant Paradigms

**DOI:** 10.3390/biom16101414

**Published:** 2026-09-29

**Authors:** João L. Afonso, Ana T. Palha, Marta F. Lima, Andreia Monteiro, Maria M. Moura, Filipa Ferreira-Antunes, Luís S. Fernandes, Sara Rito-Fernandes, Ehsan Omidvar, Inês Pereira, Beatriz Martínez-Rojas, Jonas Campos, Susana Monteiro, Belém Sampaio-Marques, Nuno A. Silva, António J. Salgado

**Affiliations:** 1 Life and Health Sciences Research Institute (ICVS), School of Medicine, University of Minho, 4710-057 Braga, Portugal; id9965@alunos.uminho.pt (J.L.A.);; 2 ICVS/3B’s-PT Government Associate Laboratory, 4805-017 Guimarães, Portugal

**Keywords:** iPSC-derived mesenchymal stem cells (iMSCs), iMSC secretome, spinal cord injury, vascular remodeling, glial modulation

## Abstract

Cell-free therapies based on the mesenchymal stem cells (MSCs) secretome are being widely explored for spinal cord injury (SCI). Induced pluripotent stem cells (iPSCs) provide a scalable source for generating mesenchymal stem cells (iMSCs), but limited studies have evaluated the modulatory capacity of iMSCs for CNS applications. Due to the importance of the glial and vascular responses to SCI progression, this study evaluated the activity of the iMSCs secretome on glial and endothelial cells in SCI-relevant paradigms. In primary mixed spinal cord cultures, the secretome was tested under supplement withdrawal and hyperosmotic injury conditions, while endothelial network formation was assessed in a morphogenesis assay. In vitro, the iMSCs secretome promoted a modest protective effect on cell survival, modulated astrocyte morphology and preserved myelin basic protein expression within the oligodendroglial lineage, without clear alterations in microglial morphometric readouts. Secretome treatment also increased *Arg1* expression in mixed spinal cord cultures. In endothelial cultures, the secretome promoted a denser and more interconnected endothelial network. In a mouse thoracic SCI model, the secretome was administered intravenously, and tissue was analyzed one week after injury. In vivo, the secretome reduced perilesional GFAP coverage and increased selected vascular morphometric parameters without altering lesion size or microglia morphological readouts. Overall, these findings indicate that the iMSCs secretome produced context-dependent changes in glial and vascular cells, mainly regarding morphological parameters, supporting further studies of iPSC-derived MSCs as a scalable source for secretome-based strategies.

## 1. Introduction

Cell-free therapies based on the mesenchymal stem cells (MSCs) secretome have been increasingly explored for central nervous system (CNS) applications due to the ability of the secretome to modulate several cellular responses, ranging from angiogenesis to neural and immunomodulation [1,2,3]. Compared with cell transplantation approaches, secretome-based strategies enable cell-free delivery and can facilitate defined dosing and formulation [4,5,6]. However, the clinical translation of the MSC-derived secretome remains constrained by donor variability, tissue heterogeneity and the limited scalability associated with primary MSC sources [7]. Induced pluripotent stem cells (iPSCs) represent an alternative source for generating MSCs with reduced heterogeneity and greater production scalability [8]. iPSC-derived MSCs (iMSCs) can be maintained for more passages with lower senescence levels, while retaining a secretome comparable to that of primary MSCs, with angiogenic, immunomodulatory and regenerative properties [8,9,10]. These features are particularly interesting in conditions where complex, multicellular responses strongly shape pathology and progression, such as spinal cord injury (SCI) [11,12].

SCI is a major cause of long-term disability and premature death, with limited endogenous repair capacity and complex interconnected secondary injury mechanisms after the initial insult [12]. Vascular and glial alterations occur early and are sustained across time, contributing to injury progression and lack of functional recovery. After injury, glial cells are the main architects behind the formation of the glial scar [13,14]. Astrocytes become reactive (hypertrophic) and surround the lesion border to limit the spread of damage into healthy tissue [14]. However, they also deposit grow-inhibitory molecules limiting axonal regrowth [15]. Microglia are also activated, migrating to the lesion and adopting a reactive (amoeboid) morphology [16]. There, they are responsible for phagocytosing cellular and myelin debris and secrete cytokines that shape the behavior of other cells. Although they can contribute to repair and protection, a persistent, non-resolving proinflammatory response exacerbates tissue damage, increasing cell death and driving injury progression [17,18]. Oligodendrocytes are also highly susceptible to secondary injury mechanisms, leading to further cell loss and demyelination, releasing debris acting as a potent axonal growth inhibitor [19,20]. Demyelinated axons are also more vulnerable to cell death and dysfunctional electrical transmission [21].

In parallel, endothelial cells are damaged after the initial insult, leading to ischemia, hemorrhage and edema that augments the secondary injury progression [22,23]. Coupled with this, the blood–spinal cord barrier is disrupted, increasing vascular permeability and facilitating uncontrolled leukocyte entry, exacerbating the damage [24,25]. Although an angiogenic response has been reported within the first week after injury, it is often insufficient or functionally immature [25,26,27].

Given the central contribution of glial and vascular cells in SCI, the effect of candidate treatments on these cells should be evaluated. For several years, MSCs have shown an ability to positively modulate these cells, mainly due to their secretome [4,5,11,28,29]. However, the effect of the iMSCs-derived secretome on glial and vascular responses remains understudied. Defining these effects, across both in vitro and in vivo paradigms, is necessary to characterize the biological activity of iMSCs.

Using iMSCs previously characterized by our group, with typical MSC morphology, expression of MSC markers CD73 and CD90 and the absence of CD45 and multilineage differentiation capacity [9], we investigated whether the iMSC secretome alters selected glial and vascular responses in experimental paradigms relevant for SCI. We evaluated the secretome effect on (i) cell survival and glial responses in mixed spinal cord cultures under supplement withdrawal and osmotic injury paradigms, (ii) endothelial network morphogenesis using human umbilical vein endothelial cells (HUVECs), and (iii) short-term glial and vascular histological responses in a thoracic SCI mouse model.

## 2. Materials and Methods

### 2.1. iPSC-Derived Mesenchymal Stem Cell (iMSC) Generation and Culture

The iMSCs used here were previously generated and were phenotypically and functionally characterized by our group [9]. iMSCs were cultured in α-MEM medium (Invitrogen, Carlsbad, CA, USA) with 5% human platelet lysate (hPL, Stemmatters, Guimarães, Portugal), 1% penicillin–streptomycin (PenStrep) (Invitrogen, Carlsbad, CA, USA) and 2 U/mL of heparin (B. Braun, Melsungen, Germany). Cells were maintained at 37 °C in a humidified chamber with 5% CO_2_, with medium changes every other day. Cell passaging was performed at 80–90% confluence, with 0.05% of trypsin–EDTA (Invitrogen, Carlsbad, CA, USA) for 5 min.

### 2.2. Secretome Production and Processing

The secretome was collected from iMSCs under the same passage (P9), following previously established protocols [30,31]. To do so, iMSCs were seeded at 4000 cells/cm^2^ and allowed to adhere and grow for 72 h. Then, cultures were washed five times with phosphate-buffered saline (PBS) without Ca^2+^/Mg^2+^ (Invitrogen, Carlsbad, CA, USA) and incubated for 24 h in serum-free conditioning medium (0.2133 mL/cm^2^). For spinal cord cultures and the SCI in vivo model, conditioning was performed in Neurobasal medium with 1% PenStrep. For endothelial assays, conditioning was performed in endothelial growth medium (R&D Systems, Minneapolis, MN, USA) with 1% PenStrep. A vehicle control consisting of the corresponding basal media was used for every experiment.

After conditioning, medium was collected, centrifuged at 250× *g* for 5 min to remove cell debris, and supernatants were snap-frozen in liquid nitrogen and stored at −80 °C.

For in vitro assays, the secretome was applied as 1× secretome (unconcentrated conditioned medium), while for in vivo studies, the secretome was concentrated 100× using centrifugal filtrations with 5 kDa molecular weight cut-off (Vivaspin, GE Healthcare, Chalfon St. Giles, UK), following previously established procedures [9,30].

### 2.3. Primary Mixed Spinal Cord Cell Cultures

Primary mixed spinal cord cultures were prepared from spinal cords of P5-P6 newborn Wistar rats (RRID_RGD_737929) of both sexes, as reported previously [31]. All animals used were treated following the local regulations of animal care and experimentation (European Union Directive 2010/63/EU), with ethical approval by the Portuguese Regulatory Entity (DGAV 022405). Briefly, the rats were euthanized by decapitation, and the spinal cord tissue was isolated by hydraulic extrusion. The spinal cords were maintained in cold sterile HBSS with 1% PenStrep until dissociation by enzymatic digestion and mechanical trituration in three rounds of 5 min each using 0.25% trypsin (Gibco, Waltham, MA, USA), at 37 °C. After this, the cell suspension was passed through a 70 µm strainer and centrifuged at 250× *g* for 10 min without brakes and then resuspended in culture medium. Cells were plated on poly-D-lysine wells at a cell density of 45,460 cells/cm^2^ (96-well plate for MTS, 48-well plates for other experiments; poly-D-Lysine incubation: 30 min at room temperature and plate drying ≥ 1 h). Plating medium consisted of DMEM-F12 (Gibco, Waltham, MA, USA) with 10% FBS (PAN Biotech, Aidenbach, Germany), 1% Glutamax (100×, Gibco, Waltham, MA, USA), 1% PenStrep and 10 ng/mL GDNF (Peprotech, Cranbury, NJ, USA). The next day, medium was changed to maintenance medium that consisted of Neurobasal medium (Gibco, Waltham, MA, USA) with 1× B27 (Gibco, Waltham, MA, USA), 1% Glutamax, 1% PenStrep and 10 ng/mL GDNF. Fresh media was changed every two days, and cells were maintained for 7 days to grow and mature before experimental analysis.

### 2.4. In Vitro Experimental Design: Supplement Withdrawal and Hyperosmotic Injury Paradigms

After 1 week of cell growth, cultures were divided into two experimental sets. In both sets, a healthy control was defined as cells growing in maintenance medium.

Set 1: Supplement withdrawal model

To study the direct effects of the secretome in spinal cord cells, medium was replaced with either vehicle medium or iMSCs secretome, both formulated without B27 and GDNF, as these factors exhibit strong trophic activity that mask secretome-induced effects. The cells were maintained up to 48 h before endpoint analysis.

Set 2: Hyperosmotic injury model

As hyperosmolarity stress is linked with several pathological processes, the effect of the secretome in the cells under osmotic stress was studied. To do so, sorbitol at 0.2 M, diluted in vehicle media, was added to the culture for 1 h, as previously established [31]. After injury, cultures received either vehicle or iMSC secretome, as in set 1.

### 2.5. Metabolic Viability Assay (MTS)

Metabolic viability was evaluated using MTS assay. After 48 h of treatment, culture media was removed, and cells were incubated in normal culture conditions with MTS reagent (Promega, Madison, WI, USA, 1:5 dilution) for 3 h. Media absorbance was read at 490 nm using a microplate reader (Varioskan, ThermoFisher, Waltham, MA, USA). Healthy controls of each independent experiment were used to normalize each experimental group.

### 2.6. Flow Cytometry

Flow cytometry was performed to assess viability and cell death population-level changes at multiple timepoints after treatment. Three timepoints were analyzed: (i) immediately after sorbitol exposure (0 h of treatment), (ii) 6 h after treatment and (iii) 24 h after treatment. At the designated timepoint, cell culture media was collected and pelleted at 250× *g* for 5 min to collect non-adherent cells and debris. In parallel, cells were detached using accutase (ThermoFisher, Waltham, MA, USA) for 5 min. Afterwards, cells were pelleted by centrifugation (250× *g*, 5 min). Both cells and cell debris were combined in binding buffer (100 mM of HEPES buffer (Merck Millipore, Rahway, NJ, USA), 140 mM of NaCl (PanReac, Castellar del Vallès, Spain) and 2.5 mM of CaCl_2_ (PanReac, Castellar del Vallès, Spain), with FITC Annexin V (BD Biosciences, Milpitas, CA, USA, 1:20) and 7-AAD (BioLegend, Milpitas, CA, USA, 1:40). Cell counting beads (BioLegend, Milpitas, CA, USA, 1:40) were added for evaluation of cell numbers. After a 15 min incubation in the dark, samples were measured in a BD LSRII flow cytometer using FACs DIVA software (BD Biosciences, Milpitas, CA, USA). Gating strategy included exclusion of debris, followed by quadrant viability classification (AnnexinV-,7-AAD-: Live cells, AnnexinV+,7-AAD-: Early apoptosis, AnnexinV+,7-AAD+: late apoptosis, AnnexinV-,7-AAD+: Dead cells.) Data was analyzed using FlowJo software (Version 10, Tree Star, Ashland, OR, USA).

### 2.7. Immunofluorescence Staining (In Vitro) and Image Analysis

Immunofluorescence was performed to quantify astrocytes, microglia and oligodendrocytes in mixed spinal cord cultures. After 48 h of vehicle or secretome incubation, cells were fixed with 4% paraformaldehyde (PFA, PanReac, Castellar del Vallès, Spain) for 15 min, followed by a PBS wash. Then, cells were permeabilized and blocked with PBS 1× with 0.1% Triton X-100 (PBS-T) and 10% newborn calf serum (NBCS, ThermoFisher, Waltham, MA, USA) for 30 min at room temperature. Primary antibodies were diluted in the same solution but with 5% NBCS and incubated overnight at 4 °C. The following antibodies were used: Rabbit anti-GFAP (1:100, Dako, Carpinteria, CA, USA), Rabbit anti-Iba-1 (1:700, Sigma-Aldrich, St. Louis, MO, USA), Mouse anti-O4 (1:500, Merck Millipore, Rahway, NJ, USA) and Rat anti-MBP (1:50, Bio-Rad Laboratories, Hercules, CA, USA). Secondary antibodies were then added in the same solution for 2 h at room temperature (Alexa Fluor^TM^ 594 Goat anti-Rabbit (1:1000, Invitrogen, Carlsbad, CA, USA), Alexa Fluor^TM^ 594 Goat anti-Rat (1:1000, Invitrogen, Carlsbad, CA, USA) and Alexa Fluor^TM^ 488 Goat anti-Mouse (1:1000, Invitrogen, Carlsbad, CA, USA)). DAPI counterstain, diluted in PBS 1× (1 µg/mL), was added for 15 min at RT. Gentle washes were done between each step.

Images were acquired using an Olympus IX81 widefield inverted microscope (Olympus, Center Valley, PA, USA), using the 20× objective. Five fields of view per well were acquired, and the data was analyzed per well.

Quantification was performed using Fiji software with 8-bit greyscale images. The DAPI signal was segmented with the Adaptive Threshold plugin, converted to a mask and processed with the watershed function. The Analyze Particles function, with size between 15–1000 and circularity ≥ 0.3, was used for cell quantification.

GFAP+ cells were segmented using Adaptive Threshold, using the standard despeckle and remove outliers processing functions, followed by positive area measurement. A DAPI-based nuclei mask was superimposed onto the GFAP image using the transparent-white function, allowing GFAP visualization with DAPI masks. For astrocyte phenotypic characterization, the cell-counter plugin was used for manual counting of the astrocytes with fibrous (fine, elongated processes) and protoplasmic (enlarged and bushy appearance) morphologies.

A similar approach was used to quantify Iba-1+, O4+ and MBP+ cells. Cells positive for O4 and MBP were manually quantified.

### 2.8. qPCR for Gene Expression

Total RNA from spinal cord cells was extracted 24 h after treatment using 1 mL of TripleXtractor (GRiSP, Porto, Portugal) per well of a 6-well plate. The reagent was added directly to the cells and incubated for 5 min, with subsequent extraction performed following the manufacturer’s instructions. RNA concentration and purity were evaluated using a NanoDrop spectrophotometer based on the A260/A280 ratio. cDNA was obtained by converting 750 ng of total RNA with Xpert CDNA Synthesis Supermix (GRiSP, Porto, Portugal). qPCR was performed using Xpert Fast SYBR Blue Mastermix with ROX reference dye (GRiSP, Porto, Portugal) in a 7500 Fast Real-Time PCR System (Applied Biosystems, Waltham, MA, USA). The primers were used at a final concentration of 500 nM each, with manufacturer’s recommended cycling conditions and an annealing temperature of 64 °C. The primer sequences are as follows: *Arg1* 5′-TCAGTGTGGTGCTGGGTGGA-3′ (forward) and 5′-GAATCCTGGTACATCTGGGAACTTT-3′ (reverse); *Gapdh* 5′-CAGTGCCAGCCTCGTCTCAT-3′ (forward) and 5′-TGGTGATGGGTTTCCCGTTGA-3′ (reverse); *Hprt* 5′-TCCCAGCGTCGTGATTAGTG-3′ (forward) and 5′-TCCAGCAGGTCAGCAAAGAAC-3′ (reverse).

Melt-curve analysis was performed to confirm amplification specificity, and a no-template control was used in each run. *Arg1* expression was normalized to the geometric mean of *Gapdh* and *Hprt* expression. Relative gene expression was calculated using the ΔΔCt method, with the CTRL group used as calibrator. Statistical analysis was performed using ΔCt values, while fold-changes were used for graphical representation.

### 2.9. Vascular Morphogenesis Assay

Human umbilical vein endothelial cells (HUVECs) were previously obtained from the umbilical cord of healthy donors, as previously reported [32]. Cells were maintained up to passage 5 in endothelial growth medium supplemented with 1× endothelial growth supplement (R&D Systems, Minneapolis, MN, USA) and 1% PenStrep, following the manufacturer instructions. For the morphogenesis assay, EHS Matrix Extract (Cell Applications Inc., San Diego, CA, USA) was polymerized in 96-well plates (40 µL/well, 30 min at 37 °C). HUVECs were passaged with 0.05% trypsin, centrifuged at 250× *g* for 5 min and resuspended in basal medium (vehicle), secretome, or supplemented endothelial growth medium before being seeded on top of the gel at 16,000 cells/well (50,000 cells/cm^2^). Cultures were then incubated for 6 h under standard culture conditions until fixed with 4% PFA for 45 min.

After permeabilization with PBS-T 0.3% for 10 min, phalloidin (0.1 µg/mL, Sigma- Aldrich, St. Louis, MO, USA) was added for 45 min to stain the actin filaments. Images were captured using the Olympus IX81 microscope (Olympus, Center Valley, PA, USA).

Quantitative morphometric analysis was performed using the Angiogenesis Analyzer plugin in Fiji [33], extracting multiple network parameters (junctions/area, meshes/area, mean mesh size, average segment length, branching interval and length density). Multivariate reduction was performed using principal component analysis (PCA) in GraphPad Prism (Boston, MA, USA). To account for inter-experimental variability, each parameter was normalized within each experiment to the mean value of the supplemented medium. All variables were centered and scaled to unit variance to ensure equal contribution of all parameters.

### 2.10. In Vivo SCI Model

All in vivo experiments were performed with approval from the responsible ethical committee (DGAV 106397.24-S) and followed the applicable regulations regarding animal care and experimentation (European Union Directive 2010/63/EU).

Adult female mice (C57BL/6J) (JAX^TM^, Charles River, Saint-Germain-Nuelles, France) were housed under controlled temperature, humidity and light conditions, with food and water provided ad libitum. Animals received daily handling 1 week prior to surgery. A total of 12 animals (10 weeks old) were randomly divided into two groups: secretome treatment (100×) or vehicle control (100×). One animal died from post-operative complications due to loss of body temperature regulation. Data collection and treatment were obtained blindly in regards to treatment group.

SCI was induced using a thoracic compression model, following previously established protocols [34,35,36]. In brief, intraperitoneal anesthesia was administered (75 mg/kg ketamine and 1 mg/kg medetomidine), and animals were shaved and disinfected with chlorhexidine. Vaseline was applied onto the eyes to prevent drying and corneal damage. A dorsal midline incision was performed at the thoracic level, and the paravertebral muscles were retracted. A laminectomy was performed at vertebral levels T8-T9 to expose the spinal cord. The spinal cord was compressed for 10 s with forceps (Dumont #5/45° angled, FST, Foster City, CA, USA). After wound closure, atipamezole was administered subcutaneously to revert anesthesia, and the animals were placed under a warm lamp.

Secretome or vehicle treatment was administered by intravenous injection at 30 min, 24 h, 48 h and 7 days post-injury (injection volume: 100 µL). Post-operative care and monitoring were performed by twice-daily subcutaneous injections of antibiotic (enrofloxacin, 5 mg/mL), analgesic (buprenorphine, 0.05 mg/kg), vitamins (Duphalyte, Pfizer, Manhattan, NY, USA) and saline (0.9% NaCl) for 5 days. Manual bladder voiding was performed two times a day. Food pellets were provided on the cage floor during the early post-operative period. Body temperature, weight, wound healing and general activity were evaluated, with humane endpoints defined for non-resolving wounds, self-injury or >20% weight loss compared to baseline. The Basso Mouse Scale (BMS) was used at 48 h post-injury to evaluate lesion effectiveness, confirmed by no motor movement in both hind paws. The animals were sacrificed at 8 days post-injury.

### 2.11. Tissue Processing and Immunohistochemistry (In Vivo)

At the defined timepoint, animals were anesthetized and transcardially perfused with 20 mL of cold PBS and 20 mL of 4% PFA. The spinal column was dissected and post-fixed for 24 h in 4% PFA. Next, the spinal cord was isolated and cryoprotected in a 30% sucrose solution until reaching saturation. Then, the tissue was cut into 1 cm fragments surrounding the injury and embedded in optimal cutting temperature solution (OCT) and frozen in isopentane and liquid nitrogen. The spinal cord was cut into coronal sections of 20 µm, mounted onto microscope slides (SuperFrost Plus) and stored at −20 °C for further use.

Immunofluorescence was performed on spinal cord sections using the following antibodies: Rabbit anti-GFAP (1:100, Dako, Carpinteria, CA, USA), Rabbit anti-Iba-1 (1:700, Sigma-Aldrich, St. Louis, MO, USA), Rat anti-CD31 (1:50, BD Biosciences, Milpitas, CA, USA). To do so, frozen sections were thawed at room temperature, washed with PBS, permeabilized with PBS-T 0.2% for 10 min and blocked for 30 min with 10% NBCS in the same solution. Primary antibodies were diluted in blocking solution but with 5% NBCS and incubated at 4 °C overnight. The next day, after washing, the samples were incubated for 3 h with the respective secondary antibodies: Alexa Fluor^TM^ 647 Goat anti-Rabbit (1:1000, Invitrogen, Carlsbad, CA, USA), Alexa Fluor^TM^ 488 Goat anti-Rabbit (1:1000, Invitrogen, Carlsbad, CA, USA) and Alexa Fluor^TM^ 594 Goat anti-Rat (1:1000, Invitrogen, Carlsbad, CA, USA). Slices were then counterstained with DAPI for 20 min before mounting the slides with Immu-Mount^®^ (ThermoFisher, Waltham, MA, USA). Images were captured using either an Olympus IX81 microscope or an Olympus FV3000 Confocal device using the Multiple Image Alignment function, with the 20× objective.

### 2.12. Immunofluorescence Analysis

Image analysis was conducted in Fiji software. Quantification was performed on 300 × 300 µm crops for better cell segmentation. The spinal cord was classified into different sections: (i) injury epicenter as the area with DAPI cluster; (ii) perilesional area defined up to 500 µm from the injury border. Analyses were focused on the epicenter and the perilesional area to assess treatment-associated effects in acute injury modulation. Rostral and caudal perilesional areas were both selected, and values were averaged to obtain one value per animal.

For GFAP staining, the adaptive threshold, with outlier removal, was used to segment the signal and create a mask for area calculation. Injury area was manually calculated via ROI drawing.

The CD31 staining mask was created using the Frangi Vesselness plugin with a scale of 2 to enhance tubular structures, followed by manual threshold and aberrant signal removal using the Analyze Particles function (size ≥ 20 and circularity ≤ 0.8). The ridge detection function was used to obtain vessel width, and a skeleton of the tubular structures was created to extract the junctions and length of CD31 staining. The CD31 mask was also superimposed on GFAP staining (with a dilation of 1 pixel) to calculate the area of vessels with an adjacent GFAP signal as a measure of GFAP+ coverage around CD31+ vessels.

The Iba-1 mask was created by subtracting the background and Gaussian blur using the autothreshold and small particle exclusion functions. Positive area was obtained, as well as amoeboid positive area, by manual classification.

### 2.13. Retrospective Proteomic Analysis of the iMSCs Secretome

A retrospective bioinformatic analysis was performed using a previously published proteomic dataset generated by Marote et al. [9]. The dataset included secretome samples of the iMSC donor used in the current experiments. Secretome collection and SWATH-MS proteomic quantification were performed as described previously. Only proteins quantified in the iMSC secretome were considered in the present analysis. Of the 107 proteins reported in the original dataset, serum albumin was excluded as a likely medium contaminant, resulting in 106 proteins being included in the analysis.

Protein–protein interaction and functional enrichment analyses were performed using STRING version 12.0. A minimum interaction confidence score of 0.7 was used, using the DBSCAN clustering method (epsilon value = 3). Gene Ontology Biological Process and Reactome enrichment analyses were used to identify the main functional categories represented within the protein list.

### 2.14. Statistical Analysis

Statistical analyses were conducted using GraphPad Version 9.4. Normality of the data distribution was assessed via the Shapiro–Wilk test and a frequency distribution histogram.

Bartlett’s tests (>2 groups) or F-tests (2 groups) were used to assess variance homogeneity. Primary statistical tests included one-way ANOVA and unpaired *t*-tests with non-parametric equivalents (Kruskal–Wallis and Mann–Whitney). Welch’s correction was used when variances differed. Post hoc corrections for multiple comparisons were applied (ANOVA: Tukey’s multiple comparisons test, Welch ANOVA: Dunnett’s T3 multiple comparisons test, Kruskal–Wallis: Dunn’s multiple comparisons test). A significance threshold of *p* < 0.05 was used (95% confidence level). Sample size and the specific statistical test are provided in each figure legend. Exact *p*-values for each statistically significant comparison can be found in Supplementary Table S1.

## 3. Results

To investigate whether the iMSCs secretome alters spinal cord glial cells in paradigms relevant for SCI, we first applied the secretome to primary mixed spinal cord cultures under two conditions. For the first condition, a supplement withdrawal model (SW), in which cultures were switched from maintenance medium (Neurobasal supplemented with B27 and GDNF; CTRL) to either vehicle (Neurobasal; SW:Veh) or the iMSC secretome (SW:Sec). Because neither treatment group exhibit B27 nor GDNF, due to their strong masking trophic effects, this set was termed the supplement withdrawal model. In the second condition, a hyperosmotic injury model was used, as previously described [31], in which cultures were exposed to 0.2 M sorbitol for 1 h, followed by incubation with Veh (Sorb+SW:Veh) or Sec (Sorb+SW:Sec) (also under supplement withdrawal conditions).

### 3.1. iMSC Secretome Effect on Cell Viability and Death

**Figure 1:**
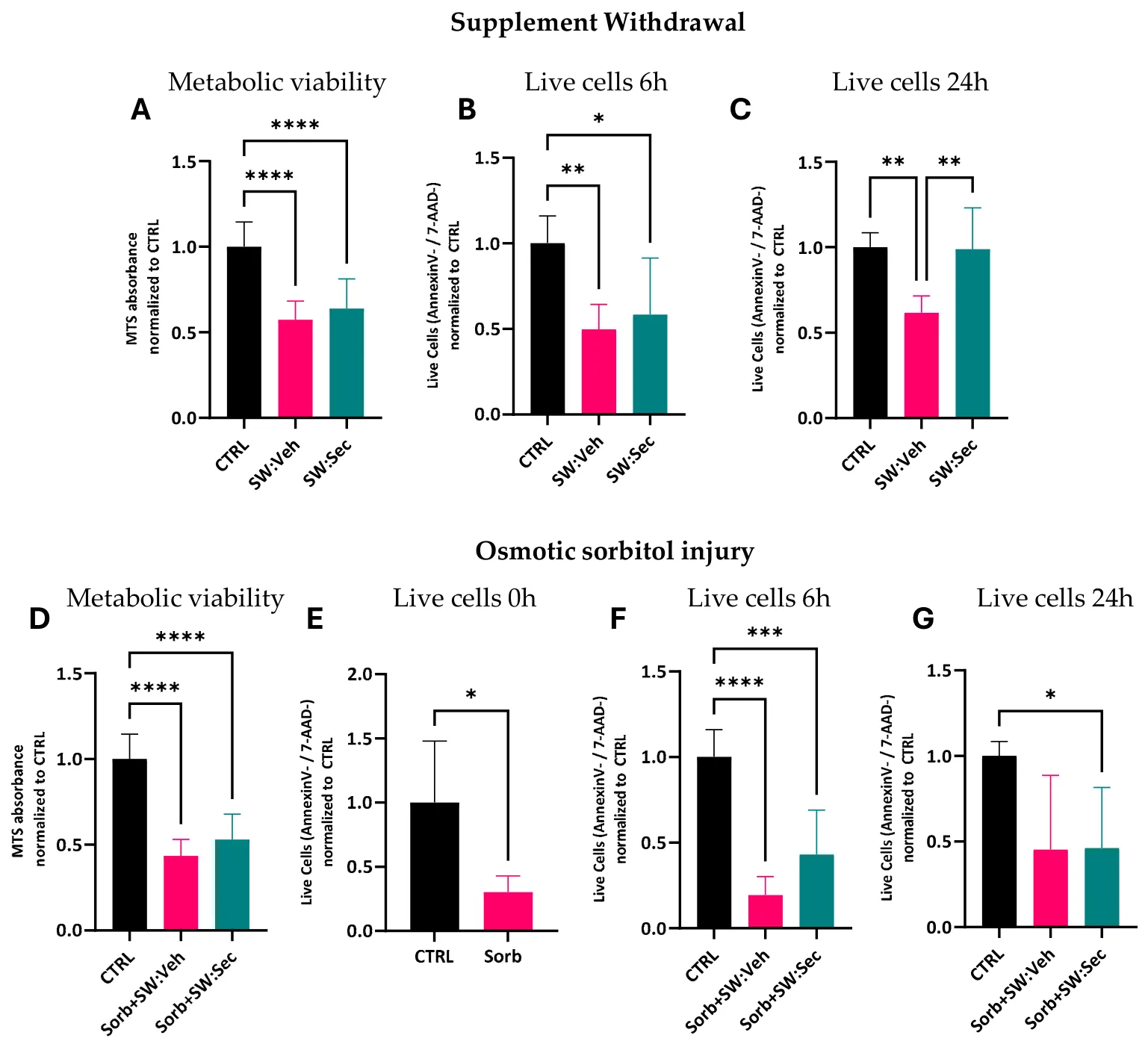
Spinal cord cell viability in supplement withdrawal (SW) and osmotic sorbitol injury (Sorb) models. (**A**) Metabolic activity quantification by MTS, 48 h after treatment, in SW model. (**B**,**C**) Flow cytometry analysis of live cells by negative staining of AnnexinV and 7-AAD in SW model. (**D**) Metabolic activity quantification by MTS, 48 h after treatment, in Sorb model. (**E**–**G**) Flow cytometry analysis of live cells by negative staining of AnnexinV and 7-AAD in Sorbitol model. CTRL: healthy cells, SW: supplement withdrawal, Veh: basal media, Sec: secretome, and Sorb: sorbitol injury. Data are shown as mean + SD. (**A**,**D**) Data is from four independent assays, with n = 4–6 wells/group per experiment. (**B**,**C**,**E**–**G**) Data is from two independent assays, each with n = 3 wells/group per experiment. All values from each experiment were normalized to the corresponding CTRL values, with well values being combined across experiments for analysis. Welch’s ANOVA was used for (**E**,**G**) and one-way ANOVA for the others. * *p* < 0.05, ** *p* < 0.01, *** *p* < 0.001, and **** *p* < 0.0001.

In order to assess a general effect of supplement withdrawal, osmotic injury and secretome treatment, the metabolic activity was evaluated by MTS at 48 h, as this was previously described as the timepoint with the highest cell death [31]. At this timepoint, MTS measurements indicated decreased metabolic activity in cultures subjected to SW relative to that of the CTRL (Figure 1A). In the hyperosmotic injury paradigm, cell viability was further decreased, as expected (Figure 1D). In both paradigms, the secretome-treated groups displayed a modest increase in cell viability compared to that of the Veh groups but still below the levels of the CTRL group.

To further evaluate this phenomenon, the dynamics of cell death were analyzed by flow cytometry using Annexin V (early apoptosis) and 7-AAD (cell death) using counting beads. In the SW set, it was possible to observe that 6 h after treatment, the number of live cells decreased in both treatment groups compared to those of the CTRL (Figure 1B). However, at 24 h, SW:Sec was able to restore the number of live cells to values similar to those of CTRL, highlighting a possible trophic/protective effect of the iMSCs secretome (Figure 1C). Following sorbitol exposure, viable cell counts were reduced immediately (Figure 1E). This effect was maintained at 6 h, with consistently higher mean values for the secretome group (Figure 1F). However, at 24 h, a higher variability in injury response was observed across experiments, with no consistent separation between vehicle and secretome conditions (Figure 1G). Overall, SW led to a decrease in live cells and cell viability that is exacerbated by sorbitol. Secretome treatment offered a modest degree of protection against this, with effects observed only on the number of live cells at 24 h under SW conditions.

### 3.2. Secretome Effects on Astrocyte Response

**Figure 2:**
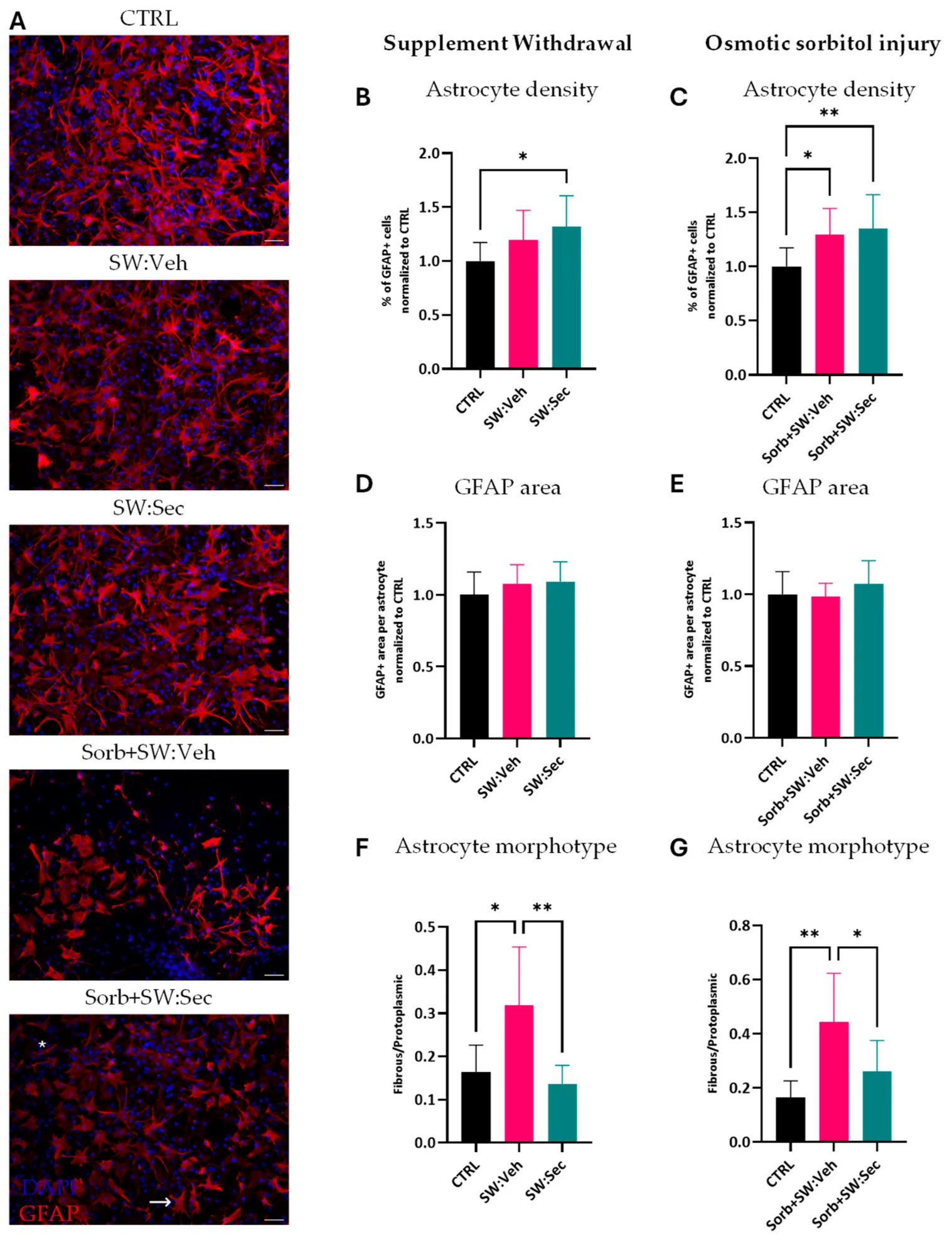
Secretome effects on astrocytic populations under cellular and osmotic stress. (**A**) Representative images of GFAP+ cells. The arrow represents protoplasmic astrocytes, while the asterisk represents fibrous astrocytes. (**B**–**E**) Quantification of percentage of GFAP+ cells relative to total DAPI+ nuclei (**B**,**C**) and mean GFAP+ area per cell (**D**,**E**), with each experiment normalized to the respective CTRL, in SW and Sorb conditions. (**F**,**G**) Quantification of morphometric changes in astrocytic population expressed as fibrous/protoplasmic ratio. Data are shown as mean + SD from three independent assays, with n = 3/4 wells/group per experiment. Five images were analyzed per well, and normalized well values were combined across experiments for analysis. (**B**–**E**) One-way ANOVA. (**F**,**G**) Welch’s ANOVA. * *p* < 0.05 and ** *p* < 0.01. Scale bar: 50 µm. Baseline CTRL values: 26.93 ± 6.70%; 783.00 ± 123.25 µm^2^.

To gain further insight into how the secretome could modulate specific glial cells under stress and injury conditions, astrocytes were examined by GFAP staining (Figure 2A). In the SW paradigm, the proportion of GFAP+ cells was increased in the secretome compared to the levels in the CTRL (Figure 2B), indicating a shift in the astrocytic representation of the cultures under secretome treatment. This was also observed under osmotic injury conditions, where astrocytic percentages were increased with both the vehicle and the secretome (Figure 2C). As total live cells decreased in these conditions, this increase may be caused by selective enrichment of the astrocytes, due to their lower vulnerability to stress, attributed to their higher metabolic flexibility and high antioxidant capacity, and not necessarily resulting from proliferation [37]. Notably, these differences were not accompanied by differences in GFAP positive area in either paradigm (Figure 2D,E). However, a morphology shift was observed in both paradigms, where the number of fibrous astrocytes relative to the number of protoplasmic types was augmented (Figure 2F,G). This alteration reverted to CTRL values via the administration of the secretome, as previously reported for primary source MSCs [31]. Altogether, these results indicate that astrocytes are responsive to cell stress and injury, and the iMSCs secretome has the ability to modulate astrocytic morphotypes.

### 3.3. Secretome Effects on Oligodendrocytes

**Figure 3:**
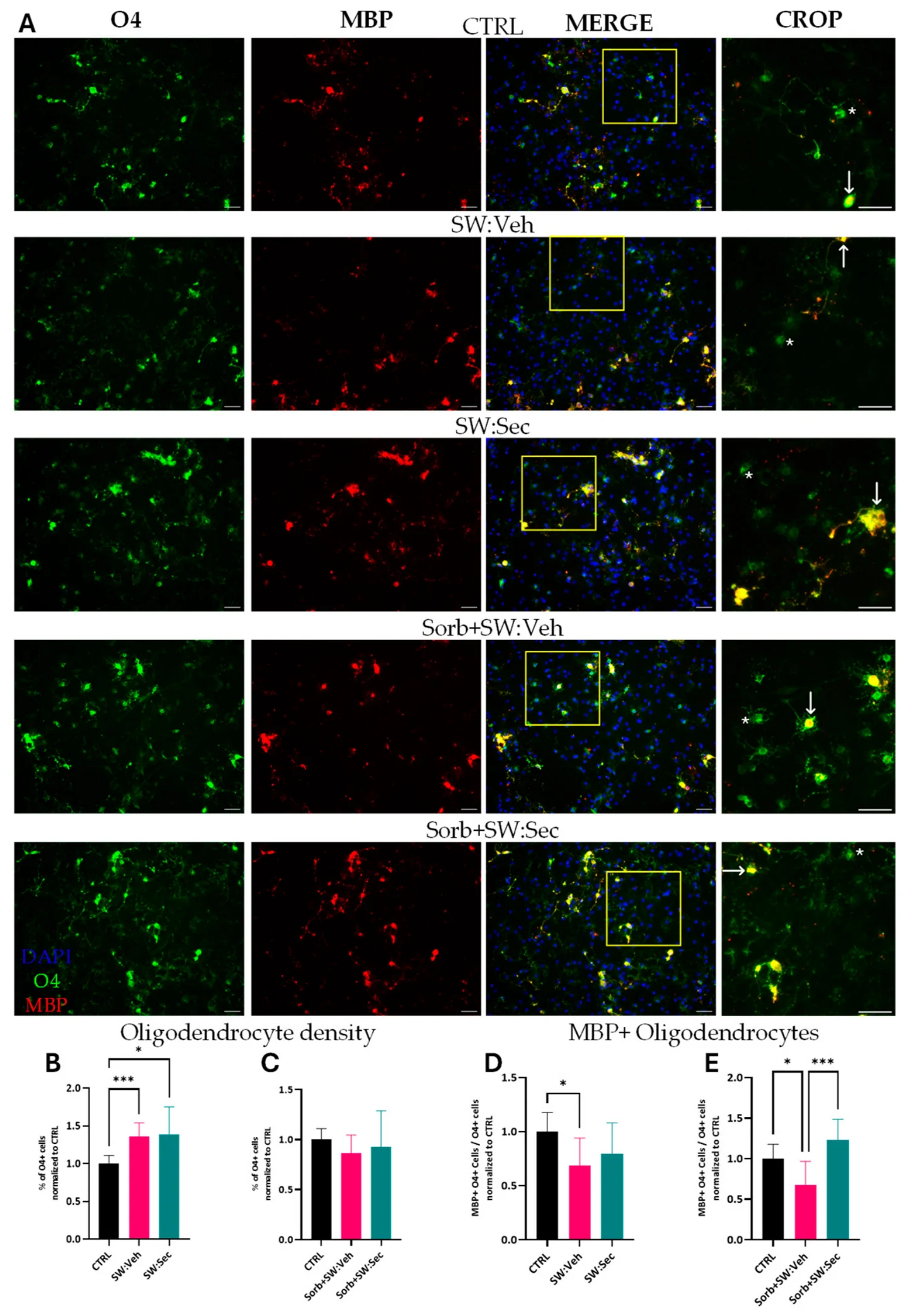
Secretome effects on oligodendrocytes under cellular and osmotic stress. (**A**) Representative microscope images of O4 and MBP+ cells, with cropped images (yellow square) highlighting O4+/MBP- (asterisk) or O4+/MBP+ (arrow) cells. (**B**,**C**) Quantification of percentage of O4+ cells relative to total DAPI+ nuclei, with each experiment normalized to CTRL, in SW and Sorb conditions. (**D**,**E**) Quantification of the percentage of MBP+ cells among O4+ cells, normalized to CTRL levels. Data are shown as mean + SD and are from three independent experiments, with n = 3/4 wells/group per experiment. Five images were analyzed per well, and normalized well values were combined across experiments for analysis. (**B**,**C**) Welch’s ANOVA. (D-E) One-way ANOVA. * *p* < 0.05 and *** *p* < 0.001. Scale bar: 50 µm. Baseline CTRL values: 8.63 ± 2.95%; 40.80 ± 9.41%.

Next, using the same paradigms, we quantified O4+ cells and MBP expression within the O4+ population by immunofluorescence to assess how the oligodendroglia lineage is modulated (Figure 3A). It was observed that, under SW, the relative proportion of O4+ cells was increased in both treatment conditions compared to that for the control (Figure 3B). In contrast, no alterations were observed under osmotic injury (Figure 3C). This may reveal that while O4+ cells are able to withstand the removal of B27 and GDNF, they are susceptible to osmotic disturbances. This was also observed at a phenotypic level, where both SW and Sorb led to a decrease in the number of MBP+ cells among O4+ cells (Figure 3D,E). While this may be the result of a direct loss of MBP expression, it could also mean a higher damage-sensibility of MBP+/O4+ cells to stress or a dysfunction in the oligodendrocyte differentiation/maturation process. Notably, the iMSCs secretome was able to revert this loss, with a stronger effect under osmotic injury conditions. This reveals that the secretome can preserve MBP in oligodendrocytes after disruption under cell stress conditions.

### 3.4. Secretome Effects on Microglial Response

**Figure 4:**
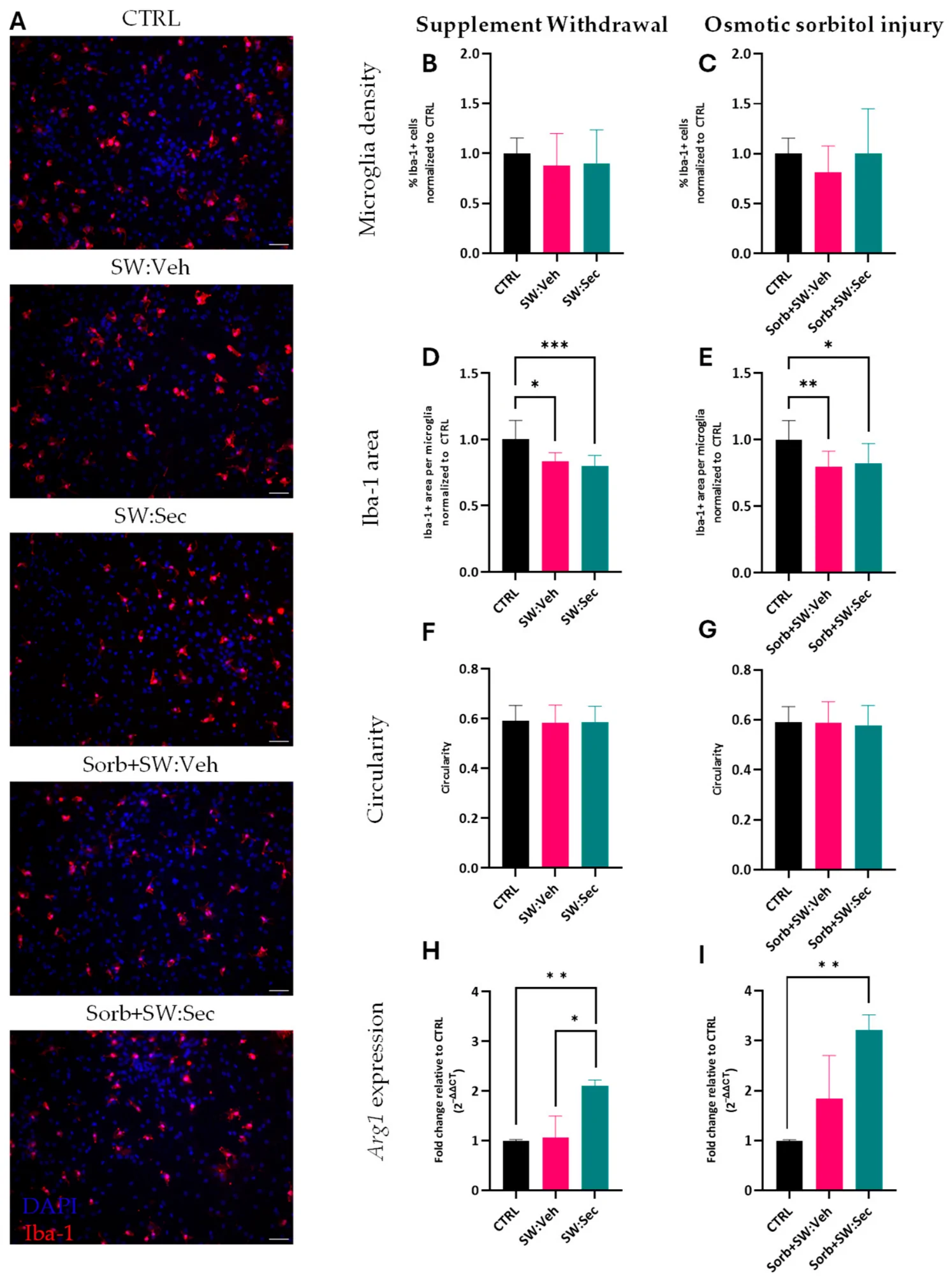
Secretome effect on microglial populations, in vitro, under cellular and osmotic conditions. (**A**) Representative pictures of Iba-1+ staining. (**B**–**E**) Quantification of percentage of Iba-1+ cells relative to DAPI+ nuclei (**B**,**C**) and Iba-1+ mean cell area (**D**,**E**), with each experiment normalized to the respective CTRL, under SW and Sorb conditions. (**F**,**G**) Circularity measurements of Iba-1+ cells. (**H**,**I**) Fold change of *Arg1* gene expression, with results normalized to CTRL. Data are shown as mean + SD and are from three independent assays, with n = 3/4 wells/group per experiment. Five images were analyzed per well, and normalized well values were combined across experiments for analysis. Gene expression is represented by geometric mean + geometric SD, with n = 3 wells. (**B**,**C**,**F**–**I**) One-way ANOVA. (**D**,**E**) Kruskal–Wallis test. * *p* < 0.05, ** *p* < 0.01 and *** *p* < 0.001. Scale bar: 50 µm. Baseline CTRL values: 27.50 ± 11.58%; 317.00 ± 163.17 µm^2^.

To elucidate how microglial cells present in the spinal cord culture are affected by stress and the secretome, cells were stained for Iba-1 (Figure 4A). No alterations were observed in the proportion of Iba-1+ cells in either paradigm (Figure 4B,C). In contrast, microglial size was reduced in SW and Sorb, with no differences in circularity (Figure 4D–G). This suggests that both conditions resulted in smaller Iba-1 cell profiles but without major morphometric shifts to more amoeboid shapes. This phenotype was also observed with secretome treatment, revealing that the secretome cannot reverse these microglial readouts. However, to complement the morphometric analysis, *Arg1* gene expression, a recognized marker for anti-inflammatory myeloid phenotypes, was analyzed. In both paradigms, secretome induced an increase in *Arg1* expression (Figure 4H,I). While not specific to microglia, this result suggests a possible inflammatory modulation by the iMSCs secretome.

### 3.5. iMSC Secretome Effect on Vascular Morphogenesis

**Figure 5:**
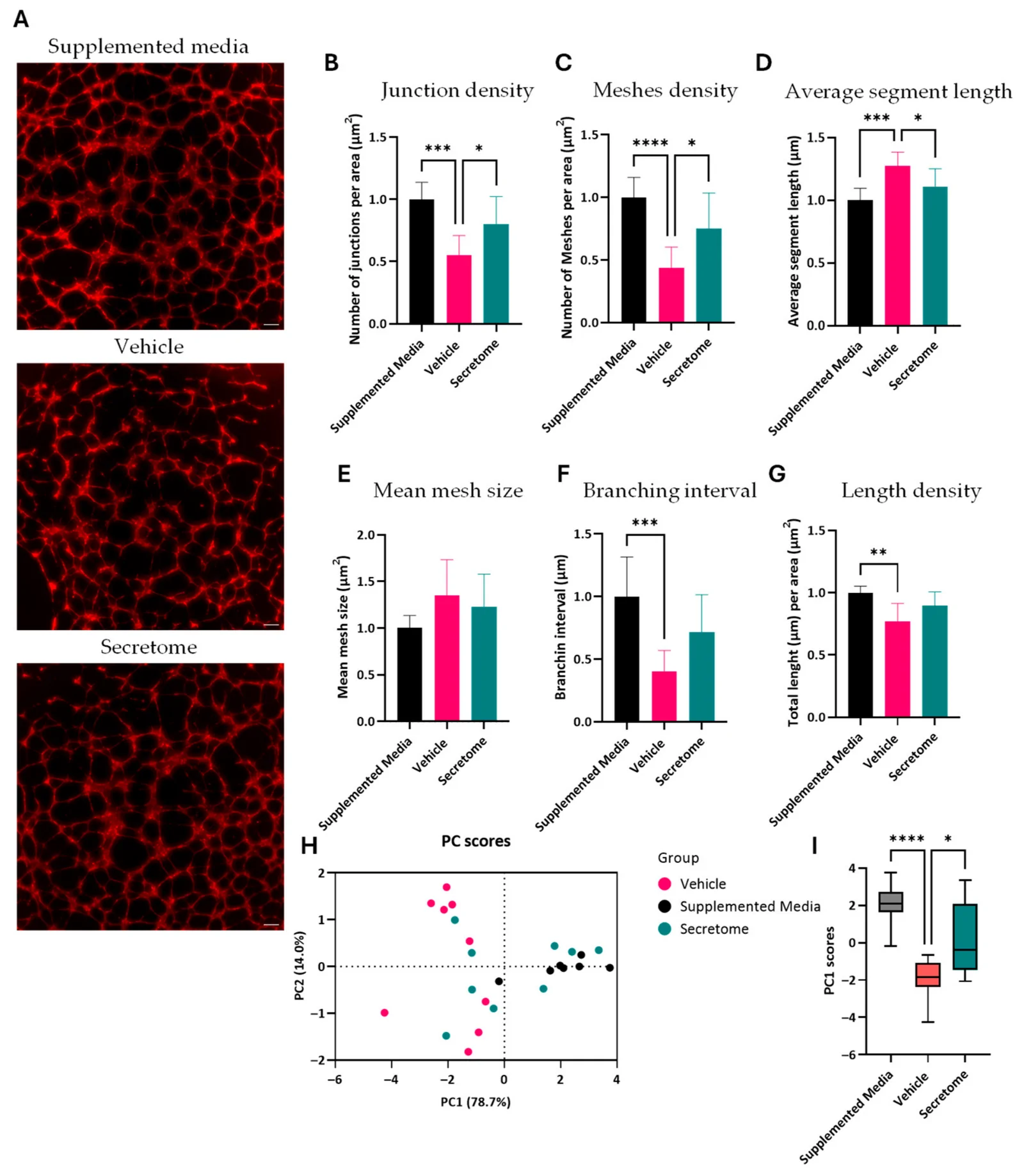
Secretome effect on HUVECs vascular organization. (**A**) Representative images of the vessel-like structures formed. (**B**–**G**) Vascular morphological parameter quantification, with each parameter normalized to the supplemented media group. (**H**) Principal component analysis score plot showing the dispersion of data points across the two first principal components. (**I**) PC1 score plot as a vascular morphogenesis index. Data shown as mean + SD in all minus I, where data is shown as min to max. Data are from three independent experiments, with n = 3 wells/group per experiment. Normalized well values were combined across experiments for analysis. One-way ANOVA * *p* < 0.05, ** *p* < 0.01, *** *p* < 0.001, and **** *p* < 0.0001. Scale bar: 200 µm.

As angiogenesis is a major process for correct tissue repair, the ability of the iMSCs secretome to influence endothelial network formation was evaluated. To do so, HUVECs were used in a vascular morphogenesis assay. In this experiment, endothelial cells were plated for 6 h in a vessel-permitting extracellular matrix, and resuspended in the secretome, vehicle or a supplemented media that induces vessel formation (Figure 5A). Relative to the supplemented media, the vehicle group produced vessel-like structures with fewer junctions and loops (meshes) and higher average segment length, revealing a less interconnected network composed of longer sparsely connected segments (Figure 5B–D). Remarkably, secretome treatment increased both junction density and meshes density, while decreasing average segment length, revealing a modulating effect on network complexity. In contrast, mean mesh size did not show a clear separation between conditions (Figure 5E). While the vehicle also produced a lower total vessel length, with a lower branching interval, the secretome did not produce a full recovery of these metrics (Figure 5F,G). Overall, across individual descriptors, the secretome improved metrics related to connectivity and complexity, with other features showing weaker or variable modulation.

Because these descriptors are functionally correlated, principal component analysis (PCA) was used to evaluate a more integrated and multi-parametric morphogenesis phenomenon. The PCA scores plot showed separation of supplemented and vehicle conditions along PC1, with secretome-treated samples occupying a sparser intermediate region (Figure 5H). Loading analysis shows that PC1 was positively correlated with branching interval, meshes density, junction density and length density and negatively correlated with average segment length and mean mesh size (Supplementary Figure S1A). PC2 captured additional variability, with mean mesh size and average segment length showing the strongest contributions, but with modest strength. In fact, these parameters were the ones that produced the most similar results between groups and might be an explanation for the wide dispersion seen across the PC2 score. Thus, higher PC1 values correspond to a phenotype with denser, more interconnected networks, and lower PC1 values correspond to network with fewer junctions and meshes but relatively longer segments with larger mesh structures. This is supported by the correlation matrix, which shows high correlation between the parameters that contribute positively to PC1 and negative or weak correlation between these factors and mean mesh size and average segment length (Supplementary Figure S1B). Based on this and because PC1 explains 78.7% of total variance, PC1 values were used as vascular morphogenesis score (Figure 5I). Consistent with the previous observations, the vehicle group produced a lower PC1 score compared to that for the supplemented media, which was increased upon secretome treatment. This supports the suggestion that the iMSCs secretome can promote a denser and more interconnected endothelial network.

### 3.6. iMSC Secretome Modulation in In Vivo SCI Context

To determine whether the iMSCs secretome retains the ability to modulate cells in an in vivo context and more specifically, in an SCI environment, a mice thoracic compression SCI model was created. The secretome or vehicle were administered intravenously, with tissue collection at 8 days post-lesion. This time window encompasses acute/subacute secondary injury events, in which the lesion is highly dynamic, characterized by an injury-associated angiogenic response and an injury core surrounded by astrocytes and immune cells that are organizing a developing glial scar. Astrocyte distribution and coverage were evaluated by GFAP staining in the epicenter and perilesional regions, corresponding to the glial scar sites. CD31 was used to evaluate the vascular features of the same compartments. Finally, microglia/macrophage responses were evaluated by Iba-1 staining. Lesion effectiveness was measured via the BMS score at 48 h, at which time all animals presented scores below 1 (Supplementary Figure S2).

### 3.7. iMSC Secretome Modulation of SCI Astrocytic Response

**Figure 6:**
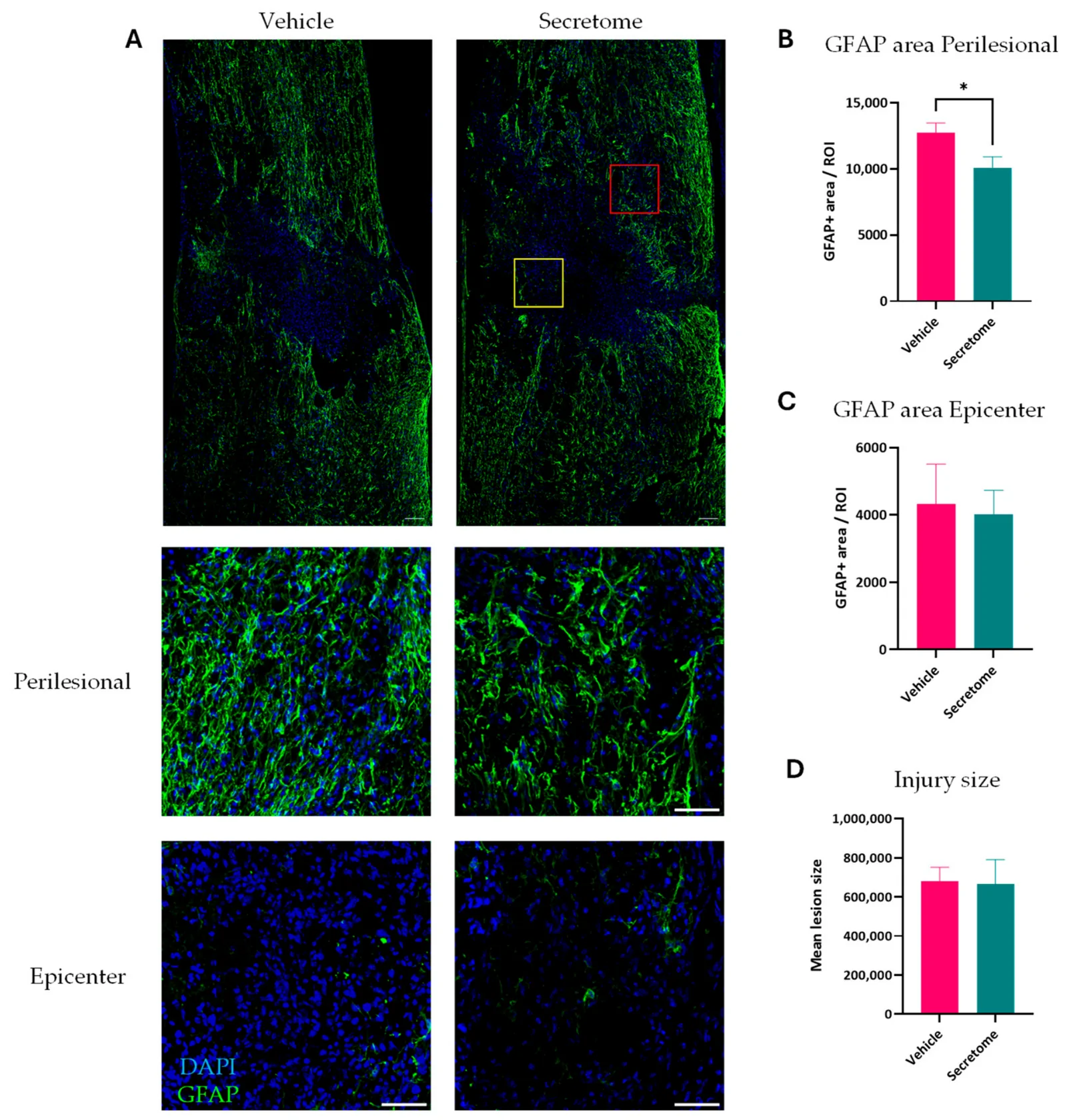
SCI astrocytic response evaluation after secretome treatment. (**A**) Representative images of the spinal cord after injury, showing the injury epicenter surrounded by GFAP+ astrocytes (green). The red square indicates the perilesional area analyzed, while the yellow square represents the epicenter region. (**B**,**C**) Quantification of the GFAP positive area in both compartments analyzed. (**D**) Measure of injury size by quantification of area inside the glial scar. Data shown as mean + SD, with n = 5/6 animals/group. Unpaired *t*-test * *p* < 0.05. Scale bar: 200 and 50 µm.

Astrocyte responses were evaluated by quantifying the GFAP area at the injury epicenter and in the perilesional border zone corresponding to the developing glial scar (Figure 6A). The GFAP signal was prominent in the perilesional compartment, consistent with astrocytic accumulation around the lesion, while the epicenter exhibited sparser GFAP staining. The secretome-treated animals displayed reduced GFAP+ area in the perilesional region compared with the levels for the vehicle, while no differences were found at the epicenter (Figure 6B,C). Furthermore, lesion size, defined by measuring the area between the rostral and caudal glial scars, was equivalent between treatments (Figure 6D). This suggests that while the secretome did not alter overall lesion size, it decreased astrocytic coverage within the scar-associated border zone at 8 days post-injury.

### 3.8. iMSC Secretome Modulation of SCI Vascular Response

**Figure 7:**
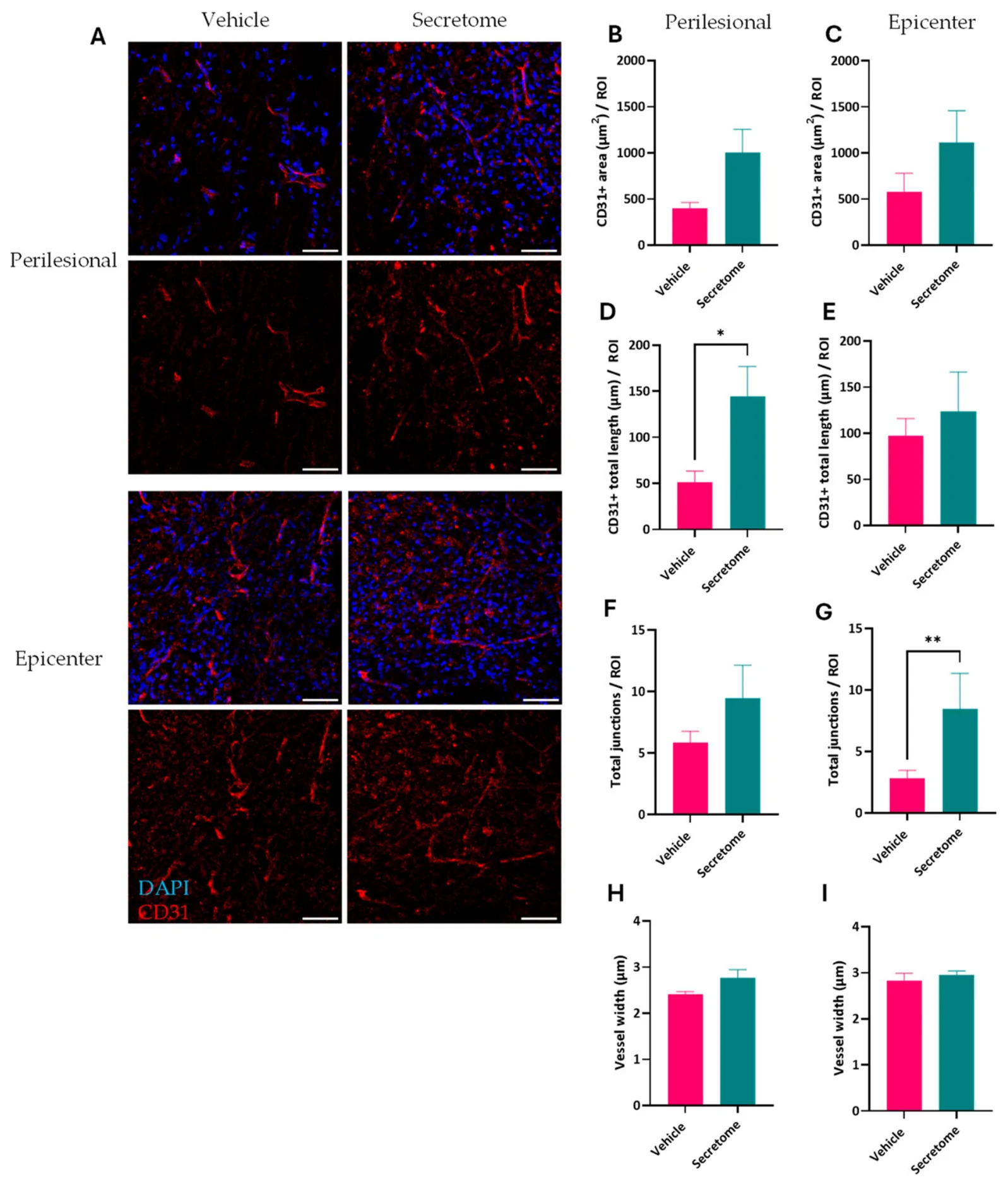
Vascular response in subacute SCI after secretome treatment. (**A**) Representative images of CD31+ cells showing vessel structures in the epicenter and perilesional areas in both groups. (**B**–**I**) Quantification of positive area, vessel total length, total junctions and vessel width in both compartments. Data shown as mean + SD, with n = 5/6 animals/group. Unpaired *t*-test * *p* < 0.05; ** *p* < 0.01. Scale bar: 50 µm.

Vascular morphology was assessed by CD31 staining, with morphometric analysis within the epicenter and the surrounding area (Figure 7A). In both regions, the secretome-treated animals exhibited higher mean values of CD31+ area (2-fold increase) but without reaching statistical significance (Figure 7B,C). However, CD31+ total length was significantly increased in the perilesional area in the secretome group compared to the results for the control (Figure 7D), indicating a higher extent of vessels in the scar-associated border zone. Some vessels were observed in the epicenter but without differences between treatments (Figure 7E). Total junctions were also significantly increased in the secretome-treated animals, with differences at the epicenter, supporting an inducing mechanism of network complexity (Figure 7F,G). No alterations were observed in the caliber of the blood vessels formed (Figure 7H,I). Furthermore, as a proxy for neurovascular unit formation, GFAP-associated blood vessels were quantified but without any differences between treatments (Supplementary Figure S3). Taken together, these data indicate that overall, secretome treatment contributes to higher vessel morphological measures, with its main effects on the vessel length in the perilesional regions and branching at the epicenter.

### 3.9. iMSC Secretome Modulation on Microglial/Macrophage Response in SCI

Microglial/macrophage responses were assessed by Iba-1 immunofluorescence in the perilesional and epicenter regions (Supplementary Figure S4A). Quantification of positive areas showed similar values between the secretome and vehicle-treated animals in both the epicenter and perilesional areas, indicating no detectable treatment effect on Iba-1 signal coverage within these regions at this timepoint (Supplementary Figure S4B,C). While all cells presented an amoeboid morphology in the epicenter, only 70% of these cells presented this morphology in the perilesional regions (Supplementary Figure S4D). Consistently, circularity measurements in either of these regions did not differ between groups (Supplementary Figure S4E,F). Together, these results indicate that this secretome regimen does not have the capacity for morphometric alteration of the microglia at this timepoint, in contrast to what was observed for the astrocytes and blood vessels.

### 3.10. iMSC Secretome Proteomic Analysis

**Figure 8:**
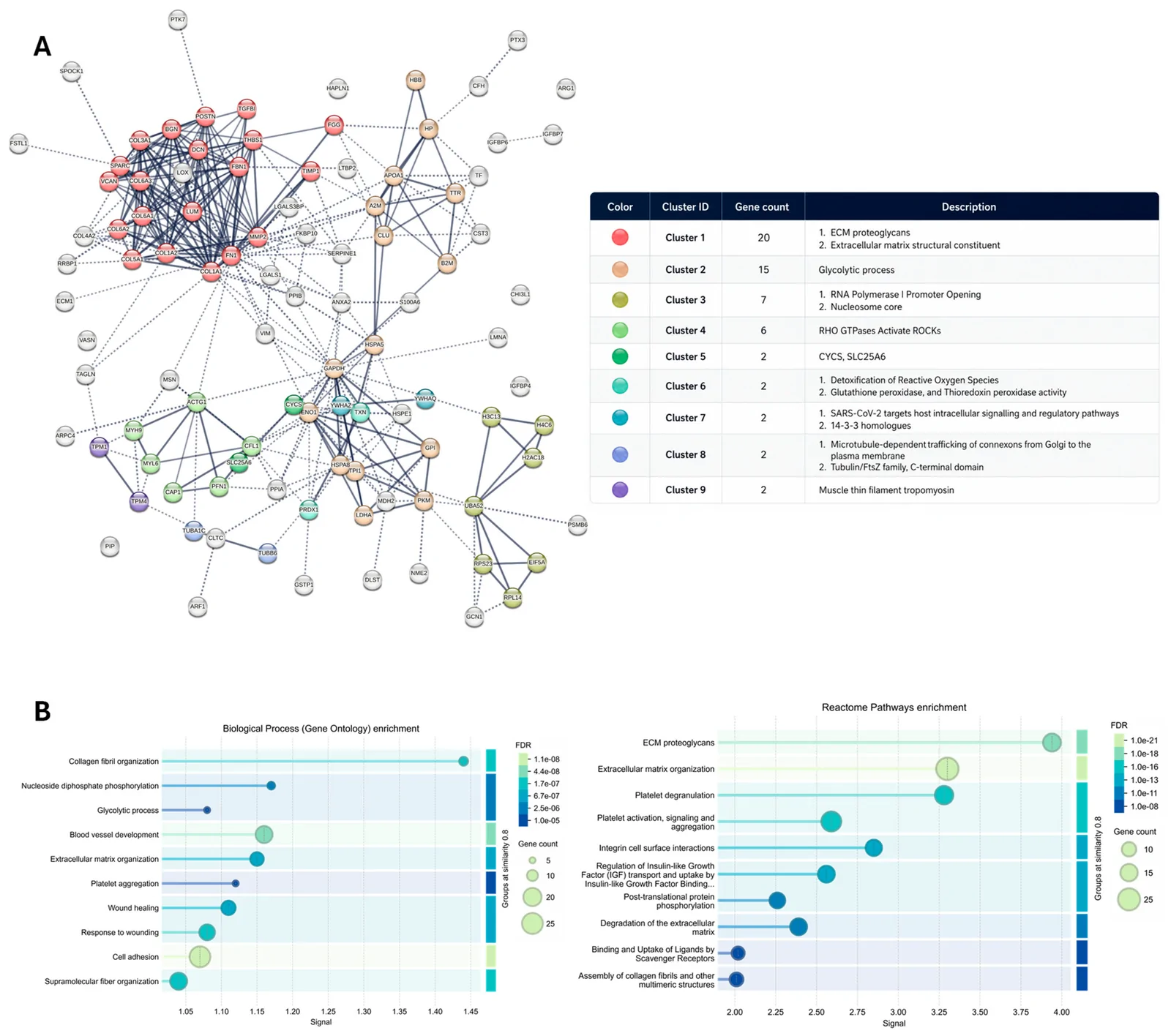
Proteomic analysis of the iMSCs secretome. (**A**) Protein–protein interaction network of iMSC secretome proteins. Node colors represent the clusters, while unclustered proteins are shown in gray. The table summarizes each cluster protein number and the main functional annotation. (**B**) Gene Ontology Biological Process enrichment and Reactome Pathway enrichment. Circle size represents the number of proteins associated with each term, circle color represents false discovery rate, and x-axis indicates the enrichment signal. Functionally related terms were grouped at a similarity threshold of 0.8.

To provide compositional context for the biological responses in this study, a previously published proteomic dataset of the iMSC secretome was retrospectively analyzed [9]. Following exclusion of serum albumin, 106 proteins were included in the STRING analysis (Figure 8A). Nine clusters were identified, of which four contained more than two proteins. The largest cluster comprised 20 extracellular matrix-associated proteins, including several collagen chains, fibronectin and proteoglycans. A second cluster contained 15 proteins associated with the glycolytic process, including glyceraldehyde 3-phosphate dehydrogenase, enolase 1, and pyruvate kinase. A third cluster identified histone and protein synthesis-associated proteins such as histone H3.2, H4, and ubiquitin-60S ribosomal protein. The fourth main cluster contained proteins associated with cytoskeletal organization, including actin and myosin.

Gene Ontology Biological Process enrichment identified collagen-related processes, extracellular matrix organization, blood vessel development, glycolytic processes, wound healing, and cell adhesion as the main represented terms. Consistently, Reactome Pathway analysis identified enrichment in terms related to extracellular matrix organization, proteoglycans, integrins and platelet signaling (Figure 8B). Overall, these data indicate that the iMSC secretome contains interconnected protein groups associated with extracellular matrix organization, glycolytic processes and cytoskeletal organization, including proteins involved in structural and adhesion-related processes that show plausible functional associations with the morphological responses observed in this work.

## 4. Discussion

The iMSC-derived secretome has been proposed as a scalable cell-free approach for tissue repair, with preclinical studies reporting modulation of inflammatory responses, improvement of osteoarthritis, attenuation of limb and cerebral ischemia, promotion of bone repair and cardioprotection [10,38,39,40,41,42]. However, the secretome effect on glial and vascular responses relevant to SCI remains insufficiently defined. In the present study, the iMSCs secretome produced context-dependent alterations on glial and endothelial cells across different in vitro paradigms, with more consistent changes in astrocyte morphology, MBP expression and endothelial network organization. The short-term in vivo findings further indicated localized changes in astrocytic and vascular parameters, suggesting that the iMSCs secretome can influence glial and endothelial responses within a complex injury environment.

To evaluate the secretome under distinct forms of cellular stress, we used primary mixed spinal cord cultures subjected to either supplement withdrawal (SW), representing a trophic restriction condition, or to sorbitol-induced hyperosmotic injury (Sorb+SW), which produces a more severe cellular insult, as reported previously [31]. SW reduced metabolic activity and the number of live cells, with these effects being further aggravated by sorbitol injury. The iMSCs secretome promoted a modest, but consistent, protective response against cell death, particularly in the SW condition, in a time-dependent manner. This protective effect was weaker and more variable after sorbitol exposure. This difference likely reflects the greater severity of the hyperosmotic insult, which may limit the capacity of the secretome-derived factors to counteract the resulting cellular damage. Overall, these findings indicate that the protective activity of the secretome depends on the nature and severity of the insult.

The iMSCs secretome also affected astrocyte parameters in the mixed spinal cord cultures. In both paradigms, the relative proportion of GFAP+ cells increased, while the overall number of cells decreased, which may be compatible with the reported resistance of astrocytes to metabolic and oxidative stress [43,44,45]. However, this may also reflect changes in the relative composition of the cultures or higher susceptibility of other cell populations. Interestingly, the most consistent secretome-associated effect was observed in astrocyte morphology, as treatment restored the stress-induced increase in the fibrous/protoplasmic ratio towards control values. While astrocyte reactivity can be characterized by hypertrophic changes [46], the morphotypes observed here more accurately reflect structural reorganization instead of reactive or inflammatory astrocytic states. To determine if astrocytic responses were also detectable in a more complex injury environment, the secretome was evaluated in a thoracic compression SCI model at 8 days post-injury. In vivo, secretome treatment was associated with a reduction in GFAP coverage in the perilesional region, without altering lesion size. Similarly, in a hypoxic–ischemic brain injury model, iMSCs and their secretome were able to reduce astrocyte reactivity in association with functional improvements [47]. Nevertheless, reduced perilesional GFAP coverage should not be directly interpreted as beneficial or detrimental, as astrocyte reactivity after SCI exerts both protective and inhibitory effects. Although the in vivo findings cannot be directly connected with the in vitro experiments, both findings indicate that the iMSCs secretome is able to elicit an astrocytic structural response in distinct experimental contexts.

Within the oligodendroglial lineage, O4+ proportions increased under SW but not after sorbitol exposure, indicating that trophic restriction and hyperosmotic injury affected this population differently. Although these data do not distinguish direct effects on oligodendroglial survival from changes in other cell populations, they are consistent with previous reports showing that osmotic alterations affect oligodendrocyte precursor cell survival in vitro [48]. While the secretome did not alter O4+ proportions, it preserved the fraction of O4+ cells expressing MBP, which was reduced in both stress conditions. This does not demonstrate oligodendrocyte maturation or functional (re)myelination, but it is an indicator that the iMSCs secretome can preserve a myelin-associated marker under cellular stress.

In contrast to the astrocytic and oligodendroglial responses, secretome treatment did not modify the measured morphometric parameters of Iba-1+ cells. However, *Arg1* expression increased following secretome treatment in both paradigms. As Arg1 is widely associated with anti-inflammatory myeloid phenotypes, this finding suggests a possible immunomodulatory response. Nevertheless, because Arg1 was measured in mixed spinal cord cultures and is not specific to microglia, it cannot be interpreted as evidence of microglial polarization. The limited morphometric response may also be related to the fact that SW and sorbitol may not provide a sufficiently strong inflammatory stimulus to induce pronounced microglial morphological changes. Similarly, in the SCI model, treatment-associated differences were not detected in Iba-1+ area and amoeboid morphology. As primary MSCs have shown immunomodulatory effects on microglia [49,50], and iMSC-based approaches are beginning to report similar microglial modulation [47], additional cytokine profiling, inflammatory markers and functional analysis, such as phagocytosis, would be required to define the specific microglial inflammatory states affected by treatment. Thus, across the present models, the effects of the iMSC secretome on microglial morphology were limited, while the Arg1 increase suggests that morphometric analysis alone does not capture the full immunomodulatory response. Overall, the iMSC secretome produced distinct context-dependent glial responses, with clearer effects on astrocyte morphology and MBP expression within O4+ cells, while the microglial response was limited at the morphometric level.

The vascular effects of the iMSCs secretome were also evaluated. MSCs are known for their strong pro-angiogenic properties, which may be relevant to induce vascular recovery after SCI [27]. In the endothelial morphogenesis assay, the iMSC secretome shifted the network organization towards a denser and more interconnected network, characterized by increased junction and mesh density and reduced average segment length. Because these descriptors represent related aspects of network organization, PCA was used to integrate the morphometric response. PC1 captured most of the variability, and its score was used as a proxy for a vascular morphogenesis index. Secretome-treated cultures presented a higher PC1 score than that of the basal media, approaching those of the supplemented media. Rather than demonstrating mature angiogenesis, these findings indicate that the iMSC secretome is able to influence endothelial network morphogenesis and organization in vitro. A similar effect was reported by Gupta et al., where iMSCs enhanced the cell viability, sprouting and tube formation of injured endothelial cells [51]. The in vivo vascular findings showed partial convergence with this endothelial response. Secretome-treated mice displayed overall higher CD31-related metrics, with significant increases in perilesional vessel length and junction number at the epicenter, while vessel width and astrocyte coverage remained unchanged. These findings are consistent with an effect on vascular organization promoted by secretome, but structural measurements alone do not establish whether these vessels were functionally perfused, mature or capable of restoring barrier functions. In fact, Menezes et al. showed that the MSCs secretome alone, despite inducing angiogenesis, was not sufficient to achieve blood vessel maturation, and that the presence of MSCs was necessary for that [52]. Thus, both in vitro and in vivo experiments support an effect of the iMSCs secretome on endothelial structural organization.

To provide additional molecular context for the biological responses observed in this study, a previously generated proteomic dataset obtained from the same iMSC source was retrospectively analyzed. The most prominent functional signature was associated with extracellular matrix organization, with enrichment of collagen-related processes, cell adhesion, blood vessel development and integrin-associated pathways. This convergence is particularly relevant because some of the clearest effects observed in the present study involved changes in cellular organization, namely astrocyte morphology and endothelial network formation. Rather than pointing to a single secreted mediator, the proteomic profile suggests an ECM–adhesion-related signature that may influence downstream cytoskeletal remodeling and cellular organization. For instance, ECM components regulate endothelial adhesion and migration, while integrin-mediated interactions can directly influence cytoskeletal organization and cell shape during vascular morphogenesis [53]. ECM composition and integrin signaling also influence astrocytic responses to injury [54]. The presence of a cytoskeletal-associated cluster provides additional structural context for the morphological responses observed here, although the detected cytoskeletal proteins themselves should not be interpreted as direct extracellular effectors. The glycolytic protein cluster may also be of biological interest, as the iMSC secretome has recently been shown to increase both glycolytic activity and mitochondrial respiration in iPSC-derived neurons [55]. However, whether the glycolysis-associated proteins identified here contribute to metabolic responses in spinal cord cells remains unknown. Overall, the proteomic data provides a plausible compositional framework in which ECM- and adhesion-related processes may contribute to the structural and morphological responses observed in this study, although further mechanistic studies are required to establish causality.

Several aspects should be considered in the interpretation of these findings. The in vitro models used here were designed to identify glial and endothelial responses to the iMSCs secretome in a fast and controlled setting. However, these models do not reproduce the full cellular, inflammatory, vascular and mechanical complexity of SCI. The subsequent in vivo experiment partially addresses this limitation by evaluating the secretome within the more complex SCI environment. Although a short-term histological timepoint was evaluated, this timepoint captures an active phase of glial and vascular reorganization following SCI. Furthermore, while histological and morphological results allow for the detection of structural responses, they do not allow the characterization of complete functional states. In addition, vascular repair involves additional processes beyond network and vessel organization, including maturation, perfusion and restoration of barrier function. Therefore, the present study should be interpreted as an investigation of how the secretome derived from a promising translational MSC source influences selected glial and vascular responses under stress and injury conditions, rather than as a demonstration of therapeutic efficacy for SCI. Longer-term studies incorporating vascular function, remyelination and behavior outcomes will be necessary to determine whether the structural changes identified here translate into sustained repair.

## 5. Conclusions

In summary, the iMSCs secretome produced biologically relevant and context-dependent effects on glial and vascular responses, with clearer alterations in astrocyte morphology, preservation of MBP expression within O4+ cells, and endothelial network organization in vitro. In the short-term SCI model, the intravenous iMSCs secretome was associated with localized alterations in astrocytic and vascular morphometric parameters. Together with the proteomic profile, these findings suggest that the modulation of cellular organization and cell–matrix interactions may represent an important component of iMSC secretome activity.

Overall, this study supports that iMSCs, as a scalable MSC source with translational potential, possess glial and vascular modulatory activity in SCI-relevant experimental contexts. These results provide a basis for further studies addressing whether these structural responses contribute to functional tissue repair after SCI.

## Data Availability

The original contributions presented in this study are included in the article/Supplementary Material. Further inquiries can be directed to the corresponding authors.

## Supplementary Materials

The following supporting information can be downloaded at: https://www.mdpi.com/article/10.3390/biom16101414/s1, Figure S1: PCA analysis loadings and correlation matrix of endothelial network organization; Figure S2: BMS score after 48 h; Figure S3: Astrocyte and blood vessel interaction; Figure S4: Secretome effect on morphology of microglia after SCI; Table S1: Exact *p*-values of statistically significant comparisons.

## Abbreviations

The following abbreviations are used in this manuscript:

7-AAD	7-aminoactinomycin D
BMS	Basso mouse scale
CNS	Central nervous system
CTRL	Control
DAPI	4′,6′-diamidino-2-phenylindole
FBS	Fetal bovine serum
GFAP	Glial fibrillary acidic protein
GDNF	Glial cell line-derived neurotrophic factor
HBSS	Hank’s balanced salt solution
hPL	Human platelet lysate
HUVECs	Human umbilical vein endothelial cells
iMSCs	Induced pluripotent stem cell-derived mesenchymal stem/stromal cells
iPSCs	Induced pluripotent stem cells
MSCs	Mesenchymal stem/stromal cells
NBCS	Newborn calf serum
PFA	Paraformaldehyde
SCI	Spinal cord injury
Sec	Secretome
Sorb	Sorbitol
SW	Supplement withdrawal
Veh	Vehicle
