# Supplementary material for: The Secretome of iPSC-Derived Mesenchymal Stem Cells Alters Glial and Vascular Responses in SCI-Relevant Paradigms
Source: Biomolecules. 2026 Sep 29;16(10):1414. doi: 10.3390/biom16101414 (PMC13702134; doi:10.3390/biom16101414)
Supplement: Supplementary file 1 [file biomolecules-16-01414-s001.zip › Supplementary Table S1.pdf]

Table S1. Exact p-values of statistically significant comparisons.

Figure	Panel	Comparison	p-value
Figure 1	A	CTRL vs SW:Veh	<0.0001
	A	CTRL vs SW:Sec	<0.0001
	B	CTRL vs SW:Veh	0.0043
	B	CTRL vs SW:Sec	0.0164
	C	CTRL vs SW:Veh	0.0022
	C	SW:Veh vs SW:Sec	0.0028
	D	CTRL vs Sorb+SW:Veh	<0.0001
	D	CTRL vs Sorb+SW:Sec	<0.0001
	E	CTRL vs Sorb	0.0150
	F	CTRL vs Sorb+SW:Veh	<.0001
	F	CTRL vs Sorb+SW:Sec	0.0004
	G	CTRL vs Sorb+SW:Sec	0.0299
Figure 2	B	CTRL vs SW:Sec	0.0169
	C	CTRL vs SW:Veh	0.0286
	C	CTRL vs SW:Sec	0.0071
	F	CTRL vs Sorb+SW:Veh	0.0177
	F	Sorb+SW:Veh vs Sorb+SW:Sec	0.0054
	G	CTRL vs Sorb+SW:Veh	0.0011
	G	Sorb+SW:Veh vs Sorb+SW:Sec	0.0297
Figure 3	B	CTRL vs SW:Veh	0.0004
	B	CTRL vs SW:Sec	0.0310
	D	CTRL vs SW:Veh	0.0326
	E	CTRL vs Sorb+SW:Veh	0.0295
	E	Sorb+SW:Veh vs Sorb+SW:Sec	0.0001
Figure 4	D	CTRL vs SW:Veh	0.0158
	D	CTRL vs SW:Sec	0.0007
	E	CTRL vs Sorb+SW:Veh	0.0060
	E	CTRL vs Sorb+SW:Sec	0.0205
Figure 5	B	Supplemented Media vs Vehicle	0.0002
	B	Vehicle vs Secretome	0.0200
	C	Supplemented Media vs Vehicle	<0.0001
	C	Vehicle vs Secretome	0.0134
	D	Supplemented Media vs Vehicle	0.0005
	D	Vehicle vs Secretome	0.0151
	F	Supplemented Media vs Vehicle	0.0005
	G	Supplemented Media vs Vehicle	0.0015
	I	Supplemented Media vs Vehicle	<0.0001
	I	Vehicle vs Secretome	0.0160
Figure 6	B	Vehicle vs Secretome	0.0410
Figure 7	D	Vehicle vs Secretome	0.0190
	G	Vehicle vs Secretome	0.0080
